# The dorsal muscle group area at the T12 vertebral level as a risk factor for tolerability of nintedanib in patients with idiopathic pulmonary fibrosis or other progressive fibrosing interstitial lung diseases

**DOI:** 10.1097/MD.0000000000038920

**Published:** 2024-07-12

**Authors:** Manabu Ono, Seiichi Kobayashi, Hanagama Masakazu, Masatsugu Ishida, Hikari Sato, Koji Okutomo, Yusuke Shirai, Kodai Takahashi, Mitsuhiro Yamada, Naoya Fujino, Shinsuke Yamanda, Masaru Yanai

**Affiliations:** a Department of Respiratory Medicine, Japanese Red Cross Ishinomaki Hospital, Miyagi, Japan; b Department of Respiratory Medicine, Tohoku University Graduate School of Medicine, Miyagi, Japan; c Department of Pulmonary Medicine, Sendai Kousei Hospital, Miyagi, Japan.

**Keywords:** dorsal muscle group area, idiopathic pulmonary fibrosis, nintedanib, progressive fibrosing interstitial lung disease

## Abstract

Nintedanib, a multi-intracellular tyrosine kinase inhibitor, reduces progression of idiopathic pulmonary fibrosis (IPF) and has been approved to use in other progressive fibrosing interstitial lung diseases (ILD) recently. However, the factors that affect the discontinuation of treatment due to adverse events is uncertain. The dorsal muscle group area at the T12 vertebral level (T12DMA) assessed on computed tomography (CT) images has been reported to be associated with mortality in chronic obstructive pulmonary disease (COPD) and other diseases. The relationship between T12DMA and the discontinuation of nintedanib remains unclear. Methods: 39 patients with IPF or other progressive fibrosing ILDs who started nintedanib at a regular dose (300 mg/day) were enrolled. We compared the characteristics between patients who stopped nintedanib at a regular dose before 6 months and/or continue to take nintedanib at a low dose (150 mg/day) and patients who were still taking nintedanib at a regular dose over 6 months. This study retrospectively investigated clinical parameters including T12DMA index (T12DMA/height^2^) to evaluate whether these parameters might serve as risk factor for the tolerability of nintedanib in patients with IPF and other progressive fibrosing ILDs. Results: Discontinuation or dose reductions of nintedanib due to adverse events were observed in 14 (35.8%) patients. A multiple logistic regression model showed T12DMA index to be the only significant risk factor for predicting for the early termination of nintedanib (odd rate, 0.549; 95% confidence interval, 0.327–0.922; *P* = .023). Conclusions: This study revealed that T12DMA index was a risk factor for the early termination of nintedanib. The initial dose of nintedanib adjusted to the differences in skeletal muscle mass and careful management of adverse events may contribute to the longer nintedanib treatment, which would lead to a better clinical outcome.

## 1. Introduction

Idiopathic pulmonary fibrosis (IPF) is a chronic, progressive fibrosing interstitial lung disease (PF-ILD) of unknown origin characterized by excessive and disordered collagen deposition and scarring of the lung parenchyma, leading to deterioration of lung function, worsening respiratory symptoms.^[1]^ The median survival time is 35 months, and the most common cause of death was acute exacerbation.^[2]^ Nintedanib is a multi-intracellular tyrosine kinase inhibitor that has anti-fibrotic properties via targeting vascular endothelial growth factor, platelet-derived growth factor, and fibroblast growth factor. Those factors are related with regulations of fibroblast proliferation and migration in the lung, which lead to the pathogenesis of IPF.^[3,4]^ Compared with placebo, nintedanib treatment resulted in a reduced annual decline in forced vital capacity (FVC), which contributed to prolonging the time to the first acute exacerbation and a survival rate in patients with IPF.^[5,6]^ Nintedanib was recommended for the treatment of IPF in the international guideline.^[7]^ Recently, nintedanib has been approved to use in other progressive fibrosing ILDs such as PF-ILD and systemic sclerosis-associated ILD (SSc-ILD).^[8,9]^ Diarrhea was the most frequent adverse event leading to treatment discontinuation in INPULSIS-ON and the INPULSIS trials.^[10]^ Several investigators have reported the tolerability of nintedanib in real-world clinical practice.^[11–13]^ According to studies limited to Japanese patients, the discontinuation rate of nintedanib at a regular dose (300 mg/day) within 6 months was approximately 40%.^[14,15]^ Discontinuations of nintedanib due to adverse events are relatively common in elderly patients (26.4%), even in younger patients (16.0%).^[16]^ Several studies reported that older age, low body weight, body mass index (BMI) were possible risk factors for discontinuation of nintedanib.^[17,18]^ We hypothesized these factors may be associated with sarcopenia, which is defined as age-related loss of skeletal muscle mass plus loss of muscle strength and/or reduced physical performance. The dorsal muscle group area at the T12 vertebral level (T12DMA) is a novel indicator to evaluate skeletal muscle using routine chest computed tomography (CT) images. The relationship between the T12DMA and the discontinuation of nintedanib remains unclear. Here we investigated predictive factors for the tolerability of nintedanib, including T12DMA, in patients with IPF and other progressive fibrosing ILDs.

## 2. Materials and methods

### 2.1. Subjects

**Figure 1.:**
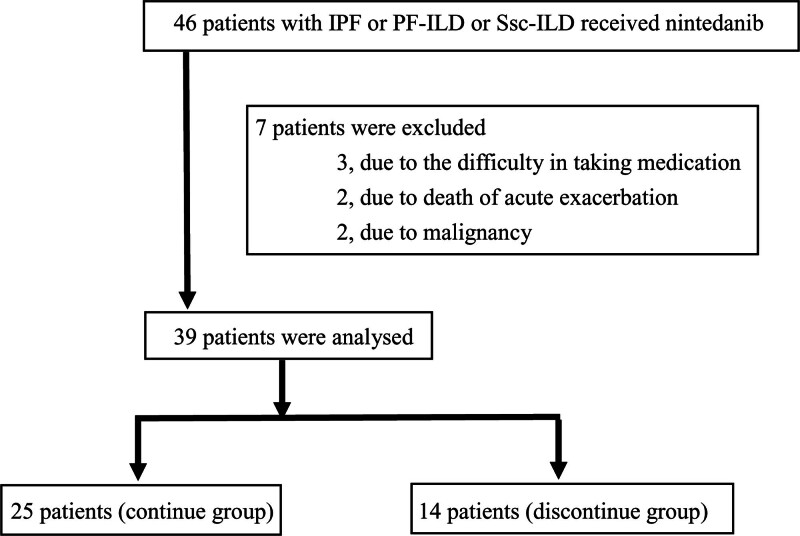
Data retrospectively collected from the patients who were diagnosed as having IPF or PF-ILD or SSc-ILD and started nintedanib at a regular dose (300 mg/d) between July 2019 and July 2023. IPF = idiopathic pulmonary fibrosis, PF-ILD = progressive fibrotic interstitial lung disease, SSc-ILD = systemic sclerosis-associated interstitial lung disease.

This retrospective study was performed at the Japanese Red Cross Ishinomaki Hospital in the Miyagi Prefecture of Japan. All included patients were diagnosed as having IPF or PF-ILD or SSc-ILD in accordance with the recent official guidelines.^[1,19,20]^ Patients with IPF, PF-ILD, SSc-ILC who started nintedanib at a regular dose (300 mg/day) between July 2019 and July 2023 were selected according to the flowchart shown in (Fig. 1). To evaluate the discontinuation of nintedanib at a regular dose within 6 months, we enrolled patients with an observation period of 6 months or more. Seven patients were excluded from this study because they were treated for acute exacerbation or died within 6 months after they started nintedanib. Patients complicated by malignant disease were also excluded, as such patients show deterioration of their general condition, making it difficult to evaluate the risk factors of discontinuation of nintedanib. This study was conducted in accordance with the 1964 Declaration of Helsinki and its later amendments or comparable ethical standards and was approved by the ethical committee of Japanese Red Cross Ishinomaki Hospital (approval number 23–21). The ethics committee deemed written informed consent not necessary due to the retrospective nature of the study. We also applied the Opt-out method to obtain consent on this study by using the poster approved by the institutional review board.

### 2.2. Data collection

We collected the baseline clinical data on age, sex, body height, body weight, body mass index (BMI), pulmonary function test including forced volume capacity (FVC) and % predicted FVC (%FVC), serum biomarker including albumin, Krebs von den Lungen-6 (KL-6) and C-reactive protein (CRP), radiological findings from the patient medical records.

### 2.3. CT analysis

**Figure 2.:**
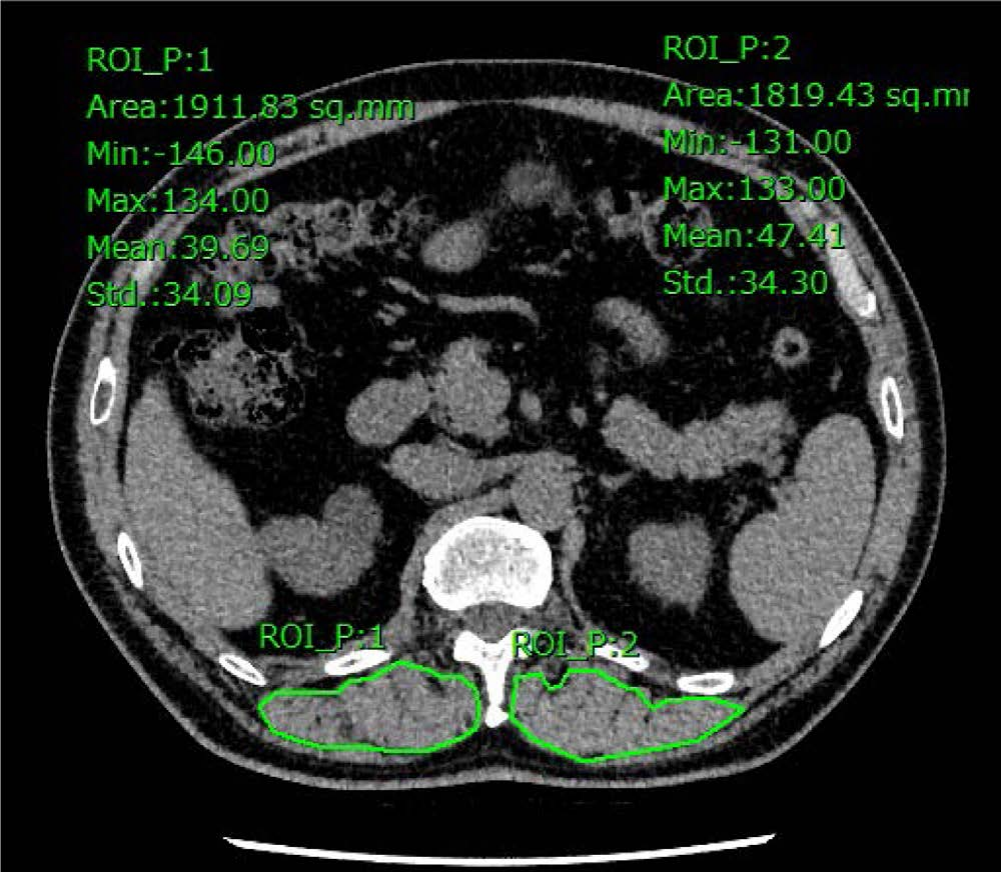
Representative picture of T12DMA. The region marked with a solid line indicates the bilateral DMA. T12DMA = T12 vertebral level.

All CT scanning was carried out before the initiation of nintedanib treatment. CT images were used to determine the quantity of skeletal muscle. The dorsal muscle group area at the T12DMA was defined as the muscle within the region at the T12 level (Fig. 2). Quantitative assessment of T12DMA was performed on a single axial slice of CT image at the level of the 12th thoracic spine and was measured with manual sketch ridge of the dorsal muscle group (DMG). T12DMA was recorded as the sum of bilateral DMG area (Table 1). Since absolute muscle mass is correlated strongly with height, we evaluated T12DMA index, as an index of relative skeletal muscle mass which was calculated as T12DMA divided by the square of the body height in meters.

**Table 1:** Characteristics of the study patients.

	Total	continue	discontinue	*P* value
	n = 39	n = 25	n = 14	
Age, yr	70.0 (64.5–78.0)	69.0 (63.0–76.0)	74.0 (68.3–79.0)	.326
Sex, male, n	31 (79.5%)	20 (80.0%)	11 (78.6%)	1.000
Female, n	8 (20.5%)	5 (20.0%)	3 (21.4%)	
Height (cm)	161.5	163.0	158.7	.312
	(155.3–169.4)	(158.0–169.5)	(152.9–165.8)	
Weight (kg)	66.9	67.0	65.4	.529
	(57.8–72.6)	(57.8–72.6)	(52.6–69.4)	
BMI (kg/m^2^)	24.7 (23.0–26.8)	24.8 (23.1–27.0)	24.7 (22.9–26.0)	.806
Albumin	3.8 (3.7–4.1)	3.8 (3.7–4.1)	3.9 (3.8–4.1)	.555
KL-6	1184	1192	1109	.784
	(906–1723)	(907–1567)	(917–2086)	
CRP	0.16 (0.07–0.33)	0.16 (0.07–0.27)	0.16 (0.11–0.41)	.618
FVC (L)	2.24 (1.76–2.85)	2.48 (1.88–2.86)	2.12 (1.65–2.50)	.213
%FVC (%)	71.8 (63.6–84.0)	71.7 (67.7–85.6)	72.5 (57.1–78.2)	.299
T12DMA (cm^2^)	25.67	26.70	23.05	.047
	(21.57–29.76)	(22.30–35.66)	(20.78–26.22)	
T12DMA index	9.84	10.30	9.00	.028
(cm^2^/m^2^)	(8.87–11.12)	(8.93–12.78)	(8.25–9.68)	
Steroid use	7 (17.9%)	3 (12.0%)	4 (28.5%)	.210
IPF	28	18	9	.478
PF-ILD	8	5	3	1.000
Ssc-ILD	3	1	2	.289

Continuous data are presented as medians (interquartile ranges) and categorical data are presented as numbers (percentages).

### 2.4. Adverse events and tolerability

Adverse events associated with nintedanib were evaluated for severity according to the common terminology criteria for adverse events version 5.0 (CTCAE ver. 5.0). The incidence of adverse events leading to dose reduction or treatment discontinuation was analyzed.

### 2.5. Statistical analysis

The patients were categorized into 2 groups; patients who stopped nintedanib at a regular dose (300 mg/day) before 6 months and/or continue to take nintedanib at a low dose (150 mg/day), classified as the “discontinued” subgroup; patients who were still taking nintedanib at a regular dose over 6 months, classified as the “continued” subgroup. Categorical data are presented as numbers (percentages), and compared using Fisher exact test. Continuous data are presented as medians (interquartile ranges), and compared using Mann–Whitney *U* test. Multivariate logistic regression analysis was performed after adjustment for factors associated with *P* value < .25 upon univariate analysis, including variables found to be of interest in previous studies (particularly age, BW). We performed all statistical analyses using EZR (Saitama Medical Center, Jichi Medical University, Saitama, Japan), which is a graphical user interface for R (The R Foundation for Statistical Computing, Vienna, Austria); *P* < .05 was considered as significant.^[21]^

## 3. Results

We enrolled 39 patients with IPF or PF-ILD or SSc-ILD who started nintedanib at a regular dose (300 mg/day) in this study. Discontinuation or dose reductions of nintedanib were observed in 14 (35.8%) patients. The baseline characteristics of the included patients and patients classified as the “continued” subgroup (n = 25) and patients classified as the “discontinued” subgroup (n = 14) are shown in Table 1. The median age of patients in the “continued” group or “discontinued” subgroup was 69.0 (range, 63.0–76.0 years) or 74.0 (range, 68.3–79.0 years), respectively. Men accounted for 80.0% and 78.6% of the 2 groups, respectively. The median BMI were 24.8 kg/m^2^ and 24.7 kg/m^2^. The median FVC at baseline were 2.48 L and 2.12 L, and %FVC were 71.7% and 72.5%, respectively. For laboratory data, there were not significant differences in levels of albumin [3.8 (3.7–4.1) vs 3.9 (3.8–4.1)] and Krebs von den Lungen-6 (KL-6) [1192 (907–1567) vs 1109 (917–2086)] and C-reactive protein (CRP) [0.16 (0.07–0.27) vs 0.16 (0.11–0.41)], respectively. 3 patients (12.0%) in the “continued” group and 4 patients (28.5%) in the “discontinued” subgroup received corticosteroid treatment for IPF or PF-ILD or SSc-ILD at baseline. Although the median T12DMA were 26.70 cm^2^ and 23.05 cm^2^ (*P* = .047), T12DMA index was lower in “discontinued” subgroup [9.00 (8.25–9.68)] than in the “continued” group [10.30 (8.93–12.78)] (*P* = .028). The most frequent adverse events that caused patients to discontinue nintedanib at a regular dose (300 mg/day) before 6 months and/or continue to take nintedanib at a low dose (150 mg/day) were diarrhea, elevation of AST, nausea, vomiting, abdominal pain (Table 2).

**Table 2:** Adverse events caused by nintedanib within 6 mo.

	Grade 1	Grade 2	Grade 3	Grade 4 or 5
Diarrhea	7 (17.9%)	1 (2.5%)	0 (0%)	0 (0%)
Elevation of AST	1 (2.5%)	7 (17.9%)	0 (0%)	0 (0%)
Nausea	3 (7.6%)	0 (0%)	0 (0%)	0 (0%)
Vomiting	1 (2.5%)	0 (0%)	0 (0%)	0 (0%)
Abdominal pain	2 (5.1%)	0 (0%)	0 (0%)	0 (0%)

Categorical data are presented as numbers (percentages).

Logistic regression analysis was performed to verify the risk factors for the early termination of nintedanib at a regular dose. Univariate analysis showed only T12DMA index to be a significant risk factor for the early termination of nintedanib (odds rate, 0.691; 95% confidence interval, 0.488–0.979; *P* = .037) (Table 3). Multivariate logistic regression analysis was performed after adjustment for factors associated with *P* value < .25 upon univariate analysis (KL-6, %FVC, T12DMA index), including variables found to be of interest in previous studies (particularly age, BW). After controlling for age and BW and KL-6, %FVC, T12DMA index, a multiple logistic regression model showed T12DMA index to be the only significant risk factor to predict for the early termination of nintedanib (odds rate, 0.540; 95% confidence interval, 0.315–0.926; *P* = .025) (Table 4).

**Table 3:** Univariate logistic regression analysis of variables.

	OR	95%CI	*P* value
Age	1.040	0.963–1.120	.318
BW	0.990	0.945–1.040	.685
BMI	0.990	0.845–1.160	.903
Albumin	0.982	0.155–6.230	.985
CRP	1.180	0.183–7.670	.859
KL-6	1.000	1.000–1.000	.119
FVC	0.458	0.166–1.260	.131
%FVC	0.969	0.927–1.010	.176
T12DMA index	0.691	0.488–0.979	.037

CI = confidence interval, OR = odds ratio.

**Table 4:** Multivariate logistic regression analysis to predict for the early termination of nintedanib.

	OR	95%CI	*P* value
Age	1.030	0.921–1.160	.578
BW	1.030	0.954–1.110	.442
KL-6	1.000	1.000–1.000	.204
%FVC	0.956	0.890–1.030	.208
T12DMA index	0.549	0.327–0.922	.023

CI = confidence interval, OR = odds ratio.

## 4. Discussion

In this retrospective study, 35.8% of patients discontinued nitedanib at a regular dose (300 mg/day) and before 6 months and/or continue to take nintedanib at a low dose (150 mg/day). The decline in paravertebral muscle size at T12DMA on chest CT images was reported to be associated with mortality in chronic obstructive pulmonary disease (COPD) and other diseases. We retrospectively investigated clinical parameters including T12DMA to evaluate whether these parameters might serve as prognostic markers for the tolerability of nintedanib in patients with IPF and other progressive fibrosing ILDs. This study revealed that T12DMA index was a risk factor for the early termination of nintedanib.

Several studies reported the tolerability of nintedanib in patients with IPF.^[11–13]^ In studies limited to Japanese patients, the discontinuation rate of nintedanib at a regular dose (300 mg/day) within 6 months was approximately 40%.^[14,15]^ Asian patients including those in Japan have higher rates of nintedanib discontinuation than patients in the USA or Europe.^[22]^ Factors including age, body weight, and Asian race have influences on the pharmacokinetics of nintedanib in patients with IPF.^[17]^ Previous data showed that patients with a smaller BSA are at higher risk of hepatotoxicity, dose reduction or discontinuation.^[23]^ Although a previous study revealed that low BMI was a risk factor for the discontinuation of nintedanib within 6 months,^[17]^ there was no significant difference in BMI between 2 groups in our study. Concerning these factors related to the pharmacokinetics and the discontinuation of nintedanib, age and low BMI were associated with sarcopenia.^[24]^ Sarcopenia might result in alterations in the distribution, metabolism and clearance of drugs, which cause increase of toxicity in patients. The Asian Working Group for Sarcopenia (AWGS) 2019 consensus defined sarcopenia as “age-related loss of skeletal muscle mass plus loss of muscle strength and/or reduced physical performance.” Furthermore, AWGS 2019 introduced the terms “possible sarcopenia,” defined as low muscle strength with or without reduced physical performance, and “severe sarcopenia” defined by low muscle mass, strength and physical performance.^[25]^ Therefore, we evaluated the skeletal muscle mass of patients, which was associated with sarcopenia.

Although the area measurement on a single cross-section CT image at the level of the third lumbar (L3) vertebra is evaluated for sarcopenia,^[26]^ abdominal CT is not a routine examination for patients with interstitial pneumoniae. Previous studies reported that the decline in paravertebral muscle size at T12DMA on chest CT images is associated with mortality in COPD and other diseases.^[27–29]^ The dorsal muscle group area at the T12DMA was used to measure the skeletal muscle mass (SMA) of patients with COPD for diagnosing sarcopenia as an alternative to L3 SMA. We hypothesized these factors may be associated with sarcopenia, therefore we used T12DMA to measure the skeletal muscle mass of patients with IPF, PF-ILD, SSc-ILD. Lean body weight (LBW) associated with sarcopenia consists of muscle and vessel-rich organs or tissues, in which drug distribution and elimination are relatively fast. The proportion of LBW is related to basal metabolic rate, drug metabolism, and clearance.^[30]^ Hence, patients with low volume of skeletal muscle mass would have high intra-tissular exposure to unbound nintedanib. According to studies about the pharmacokinetics and drug metabolism of nintedanib, when administered orally and once absorbed, nintedanib was extensively distributed in extensive tissues except in the central nervous system at steady state.^[31]^ Nintedanib showed concentration-independent plasma protein binding in the concentration range of 50 to 2000 ng/mL, with albumin being the major binding protein. The fraction of nintedanib bound to protein was 97.8% in human plasma.^[31]^ Although a potential confounding factor could be albumin, we found no relationship between albumin and the discontinuation of nintedanib at a regular dose (300 mg/day) and before 6 months.

This study had some limitations. First, the study design was a retrospective single-center study, and the sample size was small. Second, some patients were treated with steroid, which could contribute to reduction in adverse events such as nausea and vomiting. However, there was no difference of baseline characteristics between “continued” subgroup or “discontinued” subgroup. Third, the subsequent registration of adverse events was entrusted to the medical record description at the discretion of the attending physician. The reasons for discontinuation of the antifibrotic treatment varied. It is necessary to unify them and evaluate the tolerability of nintedanib prospectively.

## 5. Conclusions

In this retrospective study, 35.8% of patients discontinued nitedanib at a regular dose (300 mg/day) and before 6 months and/or continue to take nintedanib at a low dose (150 mg/day). This study revealed that T12DMA index was a risk factor for the early termination of nintedanib. The initial dose of nintedanib adjusted to the differences in skeletal muscle mass and careful management of adverse events may contribute to the longer nintedanib treatment, which would lead to a better clinical outcome.

## Author contributions

**Conceptualization:** Manabu Ono.

**Data curation:** Manabu Ono, Seiichi Kobayashi, Hanagama Masakazu, Masatsugu Ishida, Hikari Sato, Koji Okutomo, Yusuke Shirai, Kodai Takahashi, Mitsuhiro Yamada, Naoya Fujino, Shinsuke Yamanda.

**Formal analysis:** Manabu Ono.

**Investigation:** Manabu Ono.

**Methodology:** Manabu Ono.

**Project administration:** Manabu Ono.

**Resources:** Manabu Ono.

**Software:** Manabu Ono.

**Supervision:** Masaru Yanai.

**Validation:** Manabu Ono.

**Visualization:** Manabu Ono.

**Writing – original draft:** Manabu Ono.

**Writing – review & editing:** Seiichi Kobayashi.
